# Pituitary Apoplexy in Pregnancy: What do We Know?

**DOI:** 10.1055/s-0043-1770128

**Published:** 2023-06-20

**Authors:** Mariana Alves Patrício de Oliveira Gamito, Njila Yakalage Barreira Amaral, Carla Francisco Rodrigues, Joana Mariz Ribeiro, Sílvia Guerra

**Affiliations:** 1 Hospital Beatriz Ângelo, Loures, Portugal

**Keywords:** pituitary apoplexy, endocrinology in pregnancy

## Abstract

Pituitary apoplexy refers to a rare clinical syndrome consisting of signs and symptoms that occur due to rapid expansion of the contents of the *sella turcica* . It can occur spontaneously or associated with pituitary tumors. It can have a broad clinical spectrum, but usually presents with severe headache, visual impairment and hypopituitarism. Sudden onset of symptoms associated to imagiologic confirmation makes the diagnosis. Surgical treatment is advised when there is important compression of the optic tract. We present a case report and a review of the literature on pituitary apoplexy in pregnancy. The cases were reviewed to obtain information on maternal characteristics, clinical presentation, diagnostic studies, therapeutic modalities and maternal and fetal outcomes. Our review found 36 cases of pituitary apoplexy in pregnancy. Most of the cases occurred in the second trimester of pregnancy and headache was the most frequent symptom at presentation. Surgical therapy was required in more than half of the patients. In what respect maternal and fetal outcomes, there were 3 cases of preterm delivery and one case of maternal death. Our clinical case and literature review reinforces the importance of an early diagnosis to avoid potential adverse consequences.

## Introduction

Pituitary apoplexy refers to a rare clinical syndrome consisting of signs and symptoms that occur due to rapid expansion of the contents of the *sella turcica* , due to hemorrhagic or ischemic events. It can occur spontaneously or associated with pituitary tumors. In many cases, pituitary apoplexy is the initial presentation of an adenoma. The etiology is multifactorial, but several precipitating factors have been described, including pregnancy. 1 2 3 4 5 6 7

The clinical spectrum goes from asymptomatic or mild symptoms to a life-threatening situation, to both the mother and the fetus, particularly when associated with corticotropin deficiency and adrenal insufficiency. The diagnosis is made through the identification of the clinical syndrome associated with *sella turcica* imaging. Magnetic resonance imaging (MRI) is the most sensitive method to confirm the diagnosis by revealing a pituitary tumor with hemorrhagic and/or necrotic components. 4 6

The treatment of choice is conservative, but surgery might be required when there are important visual disturbances due to optic tract compression. 4 7

These study aims to report a case of a woman presenting with pituitary apoplexy during pregnancy who was treated with conservative management and also to present a review of the literature on this subject.

## Methods

We report a case of pituitary apoplexy during pregnancy and present a review of the published cases in the literature on this subject. To identify these cases, we performed a research using PubMed/MEDLINE, using the MeSH terms ‘‘pituitary apoplexy” and ‘‘pregnancy.” We included all studies published until January 2021. Our search was limited to studies published as full-text articles in English or in Portuguese. All the articles without pituitary imaging were excluded. Written informed consent was obtained from the patient described in our case report. The selected cases were reviewed to obtain information on maternal characteristics, clinical presentation, diagnostic studies, therapeutic modalities and maternal and fetal outcomes. The collected data was analyzed and summarized in a table along with the author's name and respective reference ( Chart 1 ). 1 2 3 4 5 6 7 8 9 10 11 12 13 14 15 16 17 18 19 20 21 22 23 24 25 26 27 28 29 30 31 32 33 34 35 36 37

**Chart 1:** Summary of available literature on pituitary apoplexy during pregnancy

Author [reference]	Age (years)	Clinical presentation	GA	Pituitary imaging (MRI or CT)	Treatment (GA)	Evolution	Delivery	
Prior lesion: Prolactinoma								
Tandon et al. 37	27	Headache, Visual defects	36WG	MRI: suprasellar, hemorrhagic mass with optic chiasm compression	Endoscopic endonasal transsphenoidal resection (36WG)	Resolution	C-section at term	
Castro et al. 26 -Case 2	32	Headache, nausea vomiting	28WG	MRI: intrapituitary hemorrhage	Steroids	Improvement	C-section at term	
Prior lesion: Macroprolactinoma								
Freeman et al. 8	22	Headaches, diaphoresis, Visual defects, DI	32WG	MRI: pituitary hemorrhage, with optic chiasm compression and without neurohypophysis visualization	BCP stopped when pregnancy was diagnosed Transsphenoidal evacuation	Resolution	Delivery at term	
Parihar et al. 34	22	Headache, vomiting, vision loss	20WG	Pituitary apoplexy and compression over optic nerve and chiasm	BCP stopped when pregnancy was diagnosed Transsphenoidal decompression, removal of hematoma	Resolution	Delivery at term	
Grand'Maison S et al. 15 -Case 2	30	Headache	20WG	MRI: pituitary mass with acute bleeding	Continued CBG (initiated before pregnancy)	Resolution	Vaginal delivery at term	
Jemel M et al. 20 -Case 2	35	Severe headache, nausea, vomiting deterioration of the visual ﬁeld	22WG	MRI: a pituitary mass of compatible with a pituitary adenoma in apoplexy	CBG initiated before pregnancy Microsurgical transsphenoidal		Delivery at term	
Oguz et al. 31	26	Headache, nausea, visual defects, left temporal deficit	22WG	MRI: macroadenoma with hemorrhage and optic chiasm compression	CBG Transsphenoidal surgery	Improvement	C-section at term	
Witek et al. 38	25	Headaches, dizziness, Visual defects	14WG	MRI: tumor enlargement with optic chiasm displacement and focal hemorrhage	BCP stopped when pregnancy was diagnosed Restarted BCP Transsphenoidal adenomectomy (20WG)	Improvement	C-section at term	
Gondim et al. 13	29	Headache, visual defects	30WG	MRI: Macroadenoma with inside hemorrhage	Continued BCP (initiated before pregnancy) Mini-invasive pituitary surgery (32WG)		Delivery at term	
Janssen et al. 19	27	Headache, visual defects	10WG	MRI: tumor growth, suprasellar extension and optic chiasm compression. Liquefaction within the tumor, indicating apoplexy	BCP stopped when pregnancy was diagnosed Restarted BCP	Resolution	Vaginal delivery at term	
Hayes et al. 17	41	Visual defects	18WG	MRI: pituitary hemorrhage with a signiﬁcant increase in the size of the tumor	CBG stopped when pregnancy was diagnosed Stereotactic endoscopic transsphenoidal excision (2nd trimester)		Vaginal delivery at term	
Couture et al. 6	37	Headache, Nausea, Visual defects	16WG	MRI: sellar mass with suprasellar extension and contact with the optic chiasm, compatible with hemorrhage in a pituitary tumor	BCP switched to CBG when pregnancy was diagnosed	Resolution	C-section at term	
Prior lesion: GHoma								
Lunardi et al. 24	21	Headache, Visual defects	24WG	CT: intrasellar space-occupying lesion with a marked suprasellar extension.	Transsphenoidal approach	Resolution; DI development	Normal delivery at term	
Atmaca et al. 3	33	Headache, Visual defects	33WG	MRI: Pituitary apoplexy	Transsphenoidal resection during labor		C-section at term	
Prior lesion: ACTHoma (Nelson syndrome)								
Gheorghiu et al. 12	33	Headache, nausea	22WG	MRI: intrasellar mass suggesting pituitary apoplexy		DI development	Delivery at term	
Prior lesion: Adenoma								
Ohtsubo et al. 32	29	Headache, vomiting, Visual defects	24WG	CT and MRI: pituitary adenoma with hematoma	Transsphenoidal approach (32WG)		Delivery at term	
Prior lesion: Macroadenoma								
Iuliano et al. 18	28	Headache, edema of the right optic disk	29WG	MRI: pituitary macroadenoma with hemorrhage and compression of the right optic nerve	BCP Transnasal approach (29WG)	Resolution	Delivery at term	
Unknown prior lesion								
Murao et al. [30]	35	Nausea, vomiting	39WG	MRI: pituitary apoplexy				
Kita et al. 21	26	Visual defects	26WG	MRI: pituitary mass with a ﬂuid level component displacing the optic chiasm	Endonasal endoscopic surgery (27WG)	Improvement; DI development	Delivery at term	
Krull et al. 2	28	Headache, DI	7WG	MRI: pituitary apoplexy				
Piantanida et al. 35	27	Headache, Visual defects	35WG	MRI: sellar mass with suprasellar extension, with optic chiasm compression, deviation of the pituitary stalk, and with recent bleeding	Endonasal endoscopic transsphenoidal surgery. (postpartum)	Resolution	C-section at 35WG	
Fujimaki et al. 9	23	Headache, Visual defects	24WG	MRI: large mass occupying the pituitary fossa and suprasellar cistern	Surgery was performed 1 month postpartum	Improvement	C-section at 34WG	
De Heide et al. 7	26	Headache nausea, vomiting, Visual defects, DI	23WG	MRI: pituitary tumor with hemorrhage		Improvement	Delivery at term	
Bamfo et al. 4	31	Vomiting, Visual defects, Unilateral ptosis	10WG	Hemorrhage into a preexisting solid or cystic lesion, with extension into the left cavernous sinus and optic chiasm compression			C-section at term	
Lee et al. 23	26	Headache, visual defects, low TSH and FSH and high T4, prolactin and somatomedin C	24WG	MRI: mass arising from the pituitary fossa and extending into the suprasellar cistern compressing the optic chiasm	Transsphenoidal surgery	Resolution	Vaginal delivery at term	
Grand'Maison S et al. 15 -Case 1	33	Headache, Visual defects, dizziness, neck stiffness	39WG	MRI/CT: sellar central hemorrhagic infarction and pituitary hyperplasia in contact with the optic chiasm			Labor induction at term	
Abraham RR et al. 1	32	Visual Defects decreased right V1-V2 facial	23WG	MRI: enlargement of the pituitary with hemorrhage and optic nerve compression	Emergent endoscopic endonasal surgery (23WG)			
Jemel M et al. 20 -Case 1	32	Headache, Visual defects	37WG	MRI: sellar central hemorrhagic infarction and pituitary hyperplasia, compatible with sub- acute pituitary apoplexy	None		Labor induction at term	
Jemel M et al. 20 -Case 3	30	Headache, Visual defects	24WG	MRI: bleeding within a macroadenoma	Endoscopic transsphenoidal ressection (24WG)	Improvement	Delivery at term	
Castro et al. 26 -Case 1	27	Headache, Visual defects	24WG	MRI: pituitary hemorrhage, with optic tract compression	Transsphenoidal partial excision of pituitary gland	Resolution; DI development	C-section at term	
Mathur et al. 25	34	Headache, neurological deficits, reversible vasoconstriction syndrome and stroke	Puer- perium	MRI: Pituitary apoplexy was diagnosed based on a pituitary hemorrhage	Steroids Nimodipine plus lamotrigine for seizure prophylaxis	Resolution	Emergent c- section	
Okafor et al. 33	30	Headache, vomiting. protrusion of the right eye	24WG	CT scan: pituitary tumor with pressure effects, occluding the anterior horn of the left lateral ventricle. The other ventricles were dilated	BCP	Hypertension with encephalopathy, cardiac arrest and death	Emergent C- section at 34 + 5WG	
Chan et al. 5	28	Headache, Visual defect, hypogonadism, low TSH and high serum prolactin	38WG	MRI: pituitary tumor, with hemorrhage and hypophysis and optic chiasm compression	Steroids Transsphenoidal surgery 2 days after delivery	Improvement	Forceps at term	
Galvão et al. 10 - Case 1	30	Consciousness loss, Headache, Visual defects	28WG	MRI: macroprolactinoma, with sellar and suprasellar hemorrhage	None	Resolution	C-section at term	
Galvão et al. 10 -Case 2	25	Headache, Visual defects		MRI: pituitary apoplexy	Transsphenoidal adenomectomy (2nd trimester)	Development of Hypothyroidism and DI	C-section at term	
Cokmez et al. 16	26	Headache, vomiting, Visual defects	24 WG	MRI: macroadenoma and bleeding	Steroids Tumor excision was performed with craniotomy	Improvement	C-section at term	

Abbreviations: BCP, Bromocriptine; CBG, Cabergoline; CT, computed tomography; DI, Diabetes insipidus; GA, Gestational age; MRI, Magnetic resonance imaging; WG, weeks of gestation.

## Case Report

**Fig. 1:**
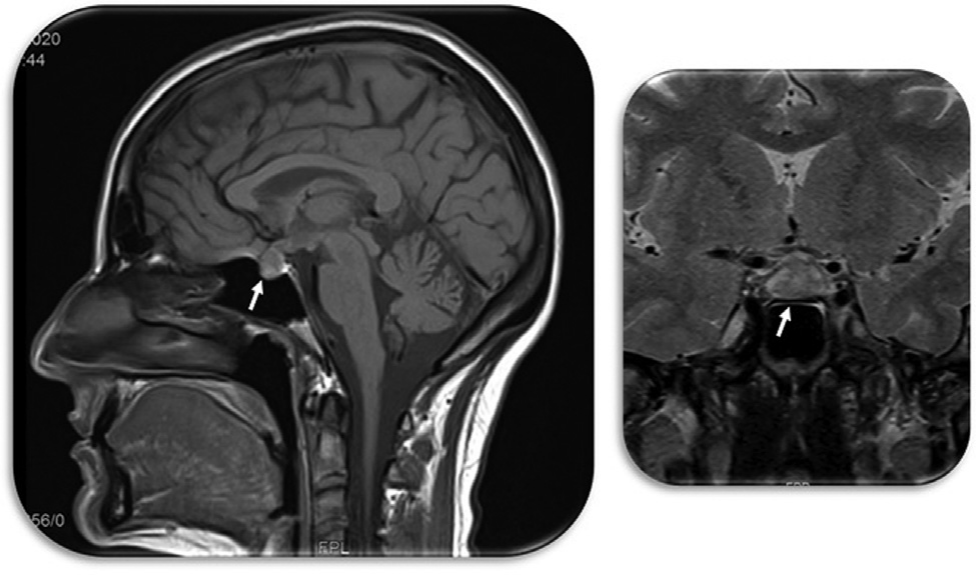
MRI showing pituitary apoplexy and arrows indicating the pituitary gland.

**Fig. 2:**
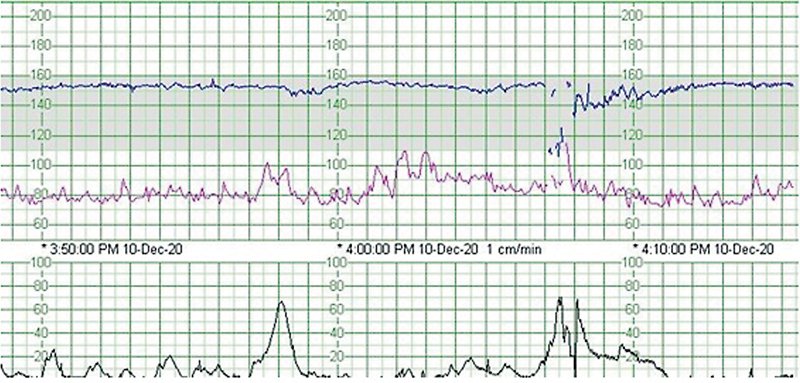
Non-reassuring fetal heart rate tracing on CTG.

A 36-year-old women, 30 weeks pregnant was admitted to the emergency service with severe holocranial headache, blurred vision, photophobia and vomiting for the last 4 days. In the day before she had been discharged from another hospital with the diagnosis of migraine. She had type 1 Diabetes Mellitus for 18 years, without known micro or macrovascular complications. She was on insulin (detemir and lispro), acetylsalicylic acid, folic acid, ferrous sulfate and potassium iodine. At admission, physical examination, blood pressure and neurologic examination were normal. Hemoglobin, platelets, renal and hepatic function were in the normal range and *sFLT-1 /PLGF* ratio was negative (<38), excluding pre-eclampsia as the cause of this clinical picture. Brain MRI was suggestive of pituitary apoplexy with compression and swelling of the optic tract: “Enlarged pituitary gland 12 mm in height, with heterogeneous sign. There is an evident suprasellar extension and shaping of the optic chiasm.” ( Fig. 1 ) After neuro-ophthalmological examination, optic chiasm compression was excluded and surgery was postponed. Blood levels of ACTH, FT4, TSH and cortisol were unremarkable. She was started on intravenous hydrocortisone 100 mg every 8 hours, with progressive improvement of symptoms. At 35 weeks of gestation, an urgent c-section was performed because of a non-reassuring fetal heart rate tracing ( Fig. 1 ) associated with absence fetal movements. A baby girl was born with 4080 g and an apgar index of 7/9. She was asymptomatic on discharge ( Fig. 2 ). In the post-partum period she remained clinically stable, asymptomatic and she was diagnosed with a non-functioning macroadenoma. She suspended corticotherapy without relapse.

## Discussion

Pituitary apoplexy is a rare event and far less frequent in pregnancy. In the absence of more robust studies, the experience provided by case reports establishes an important guidance for managing these patients. The estimated prevalence of pituitary apoplexy is 1:10000 pregnancies at term, with a mean gestational age of diagnose of 24 weeks’ gestation and 10% of cases occurring in puerperium. In many cases, as in our case report, it constitutes the first presentation of a pituitary tumor, especially macroadenomas as they tend to be more hemorrhagic. 3 14 15

Pituitary apoplexy can occur spontaneously or associated with pituitary tumors. The etiology is multifactorial, but several precipitating factors have been described: pregnancy (as in our clinical case), hemorrhagic disturbances, anticoagulation therapy, hypertension, diabetes mellitus, radiation or head trauma, cerebral aneurysm, major surgery, especially coronary artery bypass grafting, estrogen therapy, lumbar punction, upper respiratory tract infection, endocrine stimulation tests, initiation, or withdrawal of dopaminergic therapy. 1 4 5 6 7

The typical presentation of pituitary apoplexy is the one described in our case, with sudden onset of severe bilateral headache, visual disturbances, nausea and vomiting and secondary symptoms to the involvement of cranial nerves (the oculomotor is the most frequently affected). The absence of classic symptoms can delay the diagnosis. In ∼80% of the cases, patients will develop deficiency of one or more anterior pituitary hormones, depending on the percentage of pituitary tissue destroyed. Gonadotrophins are the most affected, followed by ACTH and TSH and less frequently prolactin. In this case, gonadotrophins are difficult to value since they are physiologically braked in pregnancy. 1 2 3 4

Acute secondary adrenal insufficiency is seen in approximately two-thirds of patients with pituitary tumor apoplexy and it's the major source of mortality associated with the condition, requiring prompt corticosteroid replacement in anticipation.

The diagnosis is done in the presence of the clinical syndrome associated with *sella turcica* imaging. Magnetic resonance (MRI) is the most sensitive method to confirm the diagnosis and usually reveals a pituitary tumor with hemorrhagic and/or necrotic components. 4 6

In the pregnancy context the treatment of choice is conservative. Medical therapy includes corticotherapy, dopamine agonists, such as cabergoline and bromocriptine and reposition of hormonal deficits. Surgery might be required during pregnancy, when there are important visual disturbances due to compression, endocrinal hypersecretion (especially Cushing disease) or for life-threatening apoplexy. 4 7 14

As a result of a literature research on Pubmed we found 36 case reports. From their analysis, we found that the average age of the pregnant women was 29 years old (±4 years), with an average gestational age of 25 weeks (±8 weeks) at diagnose. Most cases occurred in the second trimester. Regarding previously diagnosed lesions, it was present in 47% of patients. There were 7 cases of macroadenomas, 4 cases of microadenomas and 6 cases of adenomas without size specification. Therefore, pituitary apoplexy during pregnancy can be the first manifestation of an unrecognized pituitary adenoma in a large portion of this series. Headache was the most frequent symptom, being present in 86% of cases. Corticotherapy was used in 11% of cases and surgery was required in 61%. Most deliveries were uneventful. C-section was the mode of delivery in 15 cases, there were 6 cases of vaginal delivery and the route of delivery was unknown in 48% of the cases. There were 3 cases of preterm delivery and 28 term deliveries. There was one case of maternal death.

## Conclusion

We consider our case report an example of successful management with conservative therapy, since our patient had sustained remission of symptoms without surgery and has already suspended medical treatment without relapse. We would like to reinforce that a precocious diagnosis is essential to a timely approach, avoiding the morbimortality potentially related to this condition.
